# Supplementary material for: Merging Counter-Propagation and Back-Propagation Algorithms: Overcoming the Limitations of Counter-Propagation Neural Network Models
Source: Int J Mol Sci. 2024 Apr 9;25(8):4156. doi: 10.3390/ijms25084156 (PMC11050038; doi:10.3390/ijms25084156)
Supplement: Supplementary file 1 [file ijms-25-04156-s001.zip › Supplmentary File S1.pdf]

Supplmentary File S1

CP-ANN is based partially on an unsupervised learning - mapping of the molecules into a Kohonen (input) layer, and of a supervised learning in the Grossberg (output) layer, which represents, after it has been trained, the response surface[22-24]. The software for CP-ANN modeling was developed in-house, it is written in FORTRAN and compatible with MS Windows.

Training of the CP-ANN is divided into two consecutive steps, repeated for each new input object (molecule), until all molecules have been input:

Step 1: Identification of the most similar neuron to the input structure X_s. The neuron with the shortest Euclidean distance to the input structure is selected as the central neuron (W_c), following Eq. (1):

$$ED(X_s, W_i) = \sqrt{\sum_{j=1}^{L}(x_{sj} - w_{ij})^2} \quad (1)$$

Eq.(1) shows the Euclidean distance between the *s-th* input vector *Xs*, representing the input structure with *L* descriptors, and the neuron W_i in Kohonen neural network of dimension $Nx \times Ny$ ($i = 1 \ldots Nx \times Ny$). The dimension of each neuron is equal to the number of descriptors (i.e., each neuron has *L* components (weights)). The value of descriptor *j* of structure X_s is denoted by x_{sj}, and the value of the weight *j* in the neuron W_i is denoted by w_{ij}.

Step 2: Correction of weights in each neuron is made according to the Eq.(2).

$$w_{ij}^{(new)} = w_{ij}^{(old)} + \eta(t) f(d_c - d_i)(x_{sj} - w_{ij}^{(old)}) \quad (2)$$

In Eq.(2), ($f(d_c$-$d_i)$) is a triangular scaling function, which decreases with topological distance of neuron W_i to the central neuron W_c; $\eta(t)$ is monotonically decreasing function with increasing epochs (*t*) that depends on minimal and maximal learning rate used for training so that at the beginning $\eta(t)$ is maximal (equal to the maximal learning rate) and at the end of the training procedure it is equal to the minimal learning rate ($rate_{min} \leq \eta \leq rate_{max}$).

In the rectangular arrangement of *L*-dimensional neurons W_i, the index *i* runs from *1* to $N_x \times N_y$, which defines the two-dimensional levels of weights w_{ij}. In fact, the index *i* is split into i_x and i_y, $i_x = 1 \ldots Nx$, $i_y = 1 \ldots Ny$. The input layer can be inspected through the levels of weights. Each level corresponds to one of the *L* descriptors. The levels in the Kohonen layer are of the same dimension as the response surface in the output layer, so the distribution of individual descriptors can be compared to the responses (biological properties).

The training of the Grossberg layer uses the same equation for correction of weights as in the Kohonen layer (Eq. (2)). However, the central neuron is selected only in the Kohonen layer and then its position is projected to the Grossberg layer for the correction of weights (Eq. 2). For the supervised part of the CP-ANN learning the experimental properties (target values) of the training set objects are needed, while they are not

required for the training of Kohonen layer (unsupervised part of learning).

The above procedure (Step 1, Step 2) is repeated for all objects in the training set, which represents one epoch of the training procedure. The optimal number of epochs needed for a proper training is determined by the internal test. Either internal test set error, or the error of the leave-one-out cross validation (LOOCV) on the training set data is used for the evaluation of the model's performance during the parameter optimization.
