# Supplementary material for: Merging Counter-Propagation and Back-Propagation Algorithms: Overcoming the Limitations of Counter-Propagation Neural Network Models
Source: Int J Mol Sci. 2024 Apr 9;25(8):4156. doi: 10.3390/ijms25084156 (PMC11050038; doi:10.3390/ijms25084156)
Supplement: Supplementary file 1 [file ijms-25-04156-s001.zip › Supplmentary File S2.pdf]

Supplmentary File S2

BPE-ANN The multilayer feedforward organization of the units is the most used neural network architecture for the applications in chemistry. It consists of computational units organized into three kinds of layers, input, hidden, and output layer. The units (neurons) in each layer all receive the same information – an output vector from the previous layer, and in turn send their output vector as input to the neurons in the successive layer. The output of individual neuron is calculated as a sigmoidal function of the input signals. The units of the input layer receive their input in the form of a data file, while the units of the output layer produce the output signal, which is the overall result of the network. This multilayer architecture is often used in conjunction with the back-propagation weight update rule, according to which a supervised form of learning is implemented. The error back propagation algorithm (BPE) is essentially an iterative weight update on the basis of a steepest descent criterion, so to minimize the root-mean square error (RMS) between the desired and the actual target of the network. In mathematical terms, during the training phase each weight of the network is varied according to

$$\Delta w_{ji}(t) = -\eta \frac{\partial E}{\partial w_{ji}} + \mu \Delta w_{ji}(t-1) \qquad (3)$$

η being the learning rate and μ an additional constant called the momentum. The rightmost term, which takes into account the update of the same weight in the previous iteration (t and t-1 represent the tth and $(t\text{-}1)^{th}$ iteration respectively) has been introduced to avoid the algorithm to be stuck into local minima and to damp the oscillations of the solution.

To compute the partial derivative of the error E with respect to the connection weights to the hidden layer(s) requires propagating backwards the prediction error E using the rules of chain derivation. Hence, the name "back-propagation": chain derivation acts as a way to "distribute" the error E between the neurons of the hidden layer(s) in order to apply the iterative weight adjustment, necessary for the learning of the network.
